# Case of Aneurism of the Abdominal Aorta

**Published:** 1875-05

**Authors:** Thos. M. Mathews

**Affiliations:** Mount Enterprise, Rush County, Texas


### CASE OF ANEURISM OF THE ABDOMINAL AORTA

### SUCCESSFULLY TREATED BY LARGE DOSES OF IODIDE OF POTASSIUM.

By THOS. M. MATHEWS, M.D.,

Mount Enterprise, Rush County, Texas.

During the past winter I was called to attend Mrs. W., æt. 38, married some twelve or fifteen years, but had never had any children. I failed at the time to detect any cause for her general ill health, but in April last I discovered a large aneurism of the abdominal aorta, which on close examination I found to extend from under the sternum obliquely downward to below the umbilicus.

Having been taught that such a condition was necessarily fatal, I should have given the case up as such had I not seen the article, on page 539 of the *Amer. Journ. of the Med. Sciences* for April, 1874, by Dr. George W. Balfour, on large doses of the iodide of potassium in such cases. As a *dernier ressort* I determined to give the drug a trial. I at once put the lady upon twelve and a half grains dissolved in the syrup of sarsaparilla three times a day, and increased the dose each day till it reached fifteen grains. I then gave fifteen grains four times in the twenty-four hours. *My patient began at once to improve ;* the large doses seemed at first to irritate the stomach a little, but this soon passed away. When I began the use of the remedy the lady was confined closely to her bed ; could not sleep unless under the influence of chloral ; now, five months have passed, she is able to be up nearly all the while ; sleeps without the soporific ; rides about, even on horseback, a good deal, and is apparently in very good health. The aneurismal thrill and the bruit, once so distinct, are nearly absent, though the remains of the "sac" can be distinctly felt, yet very much smaller than it was at first. I used nothing but the iodide of potassium, except during June, when I gave, between the iodide, doses of fl. ext. ergot, ʒj, and tr. digitalis, gtt. v. I have kept up the use of the iodide steadily till now, except for two weeks ; at present I am giving iodide of potassium, gr. v, and carbonate of ammonia, gr. iij, three times a day.

Believing that in the potassic salt we have a remedy which will in a very great measure control, if not cure, this disease, which was formerly believed to be incurable, and hoping that others may be induced to try the remedy, I submit it to the consideration of the profession.

